# Advances in Electrochemical Energy Storage over Metallic Bismuth-Based Materials

**DOI:** 10.3390/ma17010021

**Published:** 2023-12-20

**Authors:** Xiaolong Cheng, Dongjun Li, Yu Jiang, Fangzhi Huang, Shikuo Li

**Affiliations:** 1 School of Material Science and Engineering, Anhui University, Hefei 230601, China; chengxl@ahu.edu.cn (X.C.); hfz@ahu.edu.cn (F.H.); 2 Department of Materials Science and Engineering, CAS Key Laboratory of Materials for Energy Conversion, University of Science and Technology of China, Hefei 230026, China; ldj0226@mail.ustc.edu.cn

**Keywords:** bismuth, electrochemical energy storage, alkali ion batteries, alkali metal anode, sulfur cathode

## Abstract

Bismuth (Bi) has been prompted many investigations into the development of next-generation energy storage systems on account of its unique physicochemical properties. Although there are still some challenges, the application of metallic Bi-based materials in the field of energy storage still has good prospects. Herein, we systematically review the application and development of metallic Bi-based anode in lithium ion batteries and beyond-lithium ion batteries. The reaction mechanism, modification methodologies and their relationship with electrochemical performance are discussed in detail. Additionally, owing to the unique physicochemical properties of Bi and Bi-based alloys, some innovative investigations of metallic Bi-based materials in alkali metal anode modification and sulfur cathodes are systematically summarized for the first time. Following the obtained insights, the main unsolved challenges and research directions are pointed out on the research trend and potential applications of the Bi-based materials in various energy storage fields in the future.

## 1. Introduction

In recent years, public opinion worldwide has been drawn toward curbing global warming and developing renewable energy. Currently, the most promising clean and renewable energy sources from solar, wind, tidal, etc., are remittent in nature and must rely on energy storage equipment to achieve full-time availability. Electrochemical energy storage devices have the advantages of short response time, high energy density, low maintenance cost and high flexibility, so they are considered an important development direction for large-capacity energy storage technology [1,2]. On the other hand, the rapidly expanding market size of various portable electronic products and electric vehicles has led to rapid growth in the demand for energy storage devices with high energy density, long lifespans, and excellent safety [3,4,5].

Among the electrochemical energy storage devices, lithium ion batteries (LIBs) have gained popularity among numerous energy storage systems owing to their high energy density, high operation potential, stable cyclability and eco-friendly nature [6,7,8]. After decades of research, LIBs have been successfully commercialized and widely penetrated into our daily lives, changing people’s lifestyles. During this development process, mobile consumer electronics products promoted the rise of the LIB industry, and the promotion of electric vehicles in recent years has directly driven the rapid growth of the LIB industry. In turn, industrialization has also increased the intensity of research, leading to the rapid development of theory and application technology for secondary batteries. Traditional LIBs with graphite as anode and layered transition metal oxides as cathode are gradually approaching their theoretical energy density, and it is difficult to further improve it [9,10]. Meanwhile, with the development of the electric vehicle industry, low global reserves of lithium resources and uneven global distribution have caused lithium salt prices to soar [11,12]. Therefore, intensive investigation has been devoted to the study of next-generation energy storage systems, including sodium ion batteries (SIBs), potassium ion batteries (PIBs), magnesium ion batteries (MIBs), lithium-sulfur (Li-S) batteries, etc. Basically, these beyond-lithium ion batteries and next-generation LIBs all operate based on the “rocking-chair” principle with alkali ions shuttling between anodes and cathodes [13,14]. Thus, what is crucial for these advanced energy storage systems is to develop appropriate electrode materials with high electrochemical performance.

**Figure 1:**
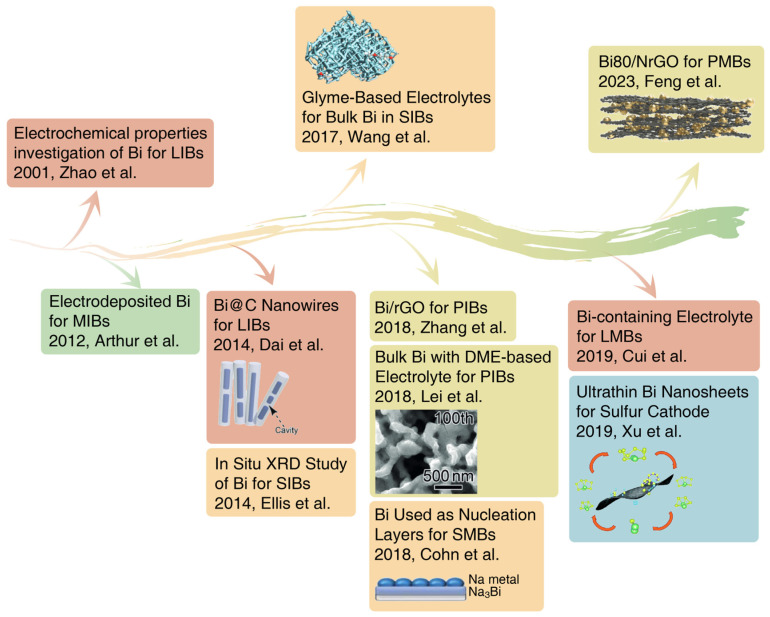
The development history of metallic Bi-based materials in electrochemical energy storage systems [21,22,24,27,28,42,47,48,49,50,51]. Reproduced with permission from Refs. [28,47,48], Copyright 2017, 2018, 2023, Wiley-VCH; from Refs. [27,49,50], Copyright 2014, 2018, 2019, The Royal Society of Chemistry.

Bismuth (Bi) is a group VA element with a unique semi-metallic nature that has been found to have good application in the photocatalysis and electrocatalysis fields [15,16,17]. Bismuth has unique electrical properties and a layered crystal structure and has been found to be promising for use in the anodes of alkali ion batteries, mainly due to its high theoretical volumetric specific capacity and appropriate operating potential [18,19,20]. Compared with other alloying anodes, such as Si, Sn, Sb and Ge, Bi has higher theoretical volumetric capacity of 3800 mAh cm^−3^. Zhao et al. demonstrated the application prospects for bismuth-based materials in LIBs and revealed the existence of binary Li-Bi alloys via ex situ XRD analyses in 2001 [21]. Later, owing to its high specific capacity, bismuth was explored as an anode material for MIBs, SIBs and PIBs in 2012, 2014 and 2018, respectively (Figure 1) [22,23,24]. 

However, the large volume variation during the alloying/dealloying process is the main reason hindering the application of Bi-based anodes for alkali ion batteries [25,26]. Severe volume expansion directly destroys the structure of the electrode material, causing the electrode to be pulverized and fall off, which in turn causes the capacity of batteries to decay rapidly. To solve these problems, massive efforts have been devoted to Bi-based anodes by downsizing the particle size, designing innovative architectures and optimizing electrolyte configurations. For example, Dai and coworkers constructed Bi@C core-shell nanowires in 2021, with the carbon shell accommodating large volume expansion and efficiently confining the nanowires [27]. Wang et al. proposed ether-based electrolytes for bulk Bi anode, which has interaction effects completely different from traditional ester-based electrolytes, and greatly promoted the advance of Bi-based anode materials [28].

For the purpose of breaking through the upper limit of traditional lithium-ion batteries, lithium metal batteries (LMBs) were re-proposed [29,30]. Based on similar considerations, sodium metal batteries (SMBs) and potassium metal batteries (PMBs) stand out among beyond-lithium ion batteries [31,32]. However, these battery systems with alkali metal anodes suffer from poor cycling stability because of the high reactivity of alkali metal, unlimited volume variation and dendrite growth [33,34,35]. For these issues, several strategies have been proposed to stabilize the alkali metal anodes, including constructing 3D hosts [36,37], metal–electrolyte interfacial engineering [38,39] and electrolyte optimization [40,41]. Inspired by the research progress on Bi-based anodes in alkali ion batteries, Bi-base materials were introduced to solve these key scientific issues for alkali metal anodes. For example, Cui et al. used K_4_BiI_7_ as an electrolyte additive for LMBs, which generated a robust and Li-ion-conductive solid electrolyte interphase (SEI) [42]. The Bi-containing electrolyte significantly improved the average coulombic efficiency and relieved Li dendrite growth. 

Li-S batteries with sulfur as cathodes have the characteristics of high specific capacity, abundance and nontoxic active material, which is considered as the best choice for next-generation LIBs [43,44]. Unfortunately, the shuttle effect of the polysulfides and slow reaction kinetics restrict its practical performance. According to the reported researches, an ideal sulfur host must provide strong surface polarity, prominent electrocatalytic activity and large specific surface area [45,46]. Noteworthily, the semi-metallic nature and the typical 2D layered crystal structure of Bi make it viable as an electrocatalyst for the conversion reaction of polysulfides. Metallic Bi-based materials have shown great progress in recent years; thus, a systematic review of the investigations of metallic Bi-based materials in different energy storage systems is imperative to summarize existing research progress and anticipate future development directions.

Herein, we summarize the recent advances in metallic Bi-based materials in the field of electrochemical energy storage systems, as shown in Figure 1. The modification strategies and innovative application of metallic Bi-based materials are discussed comprehensively. On the other hand, and more importantly, this review provides a better understanding of Bi-based materials and corresponding detailed reaction mechanisms in various battery systems. Moreover, the pressing challenges and opportunities of Bi-based materials in electrochemical energy storage systems are also summarized and discussed. This review closes with the outlook for the efficient design of Bi-based materials and research directions for excellent electrochemical performance. 

## 2. Fundamentals of Bismuth

### 2.1. Crystal Structure of Bi

**Figure 2:**
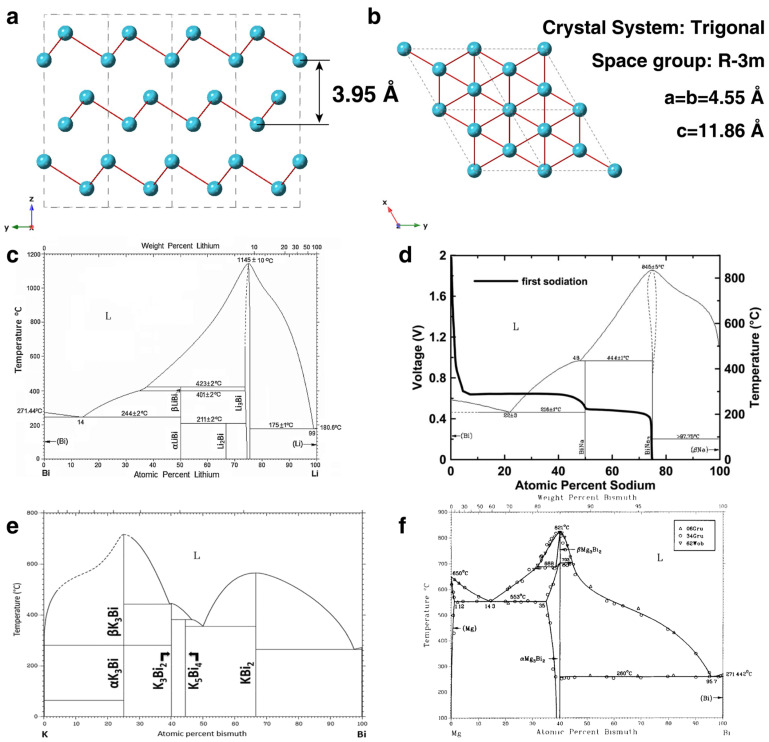
(**a**,**b**) The crystal structure of Bi. (**c**) The phase diagram of Li-Bi alloy. Reproduced with permission [53]. Copyright 2015, Springer Nature. (**d**) The phase diagram and discharge curves of Na-Bi alloy. Reproduced with permission [51]. Copyright 2014, IOP Publishing. (**e**) The phase diagram of K-Bi alloy. Reproduced with permission [54]. Copyright 2018, American Chemical Society. (**f**) The phase diagram of Mg-Bi alloy. Reproduced with permission [55]. Copyright 1985, Springer Nature.

Bi is the last element in the pnictogens and is considered as a nontoxic and environment-friendly metal. The crystal structure of Bi is rhombohedral symmetry, which is typical for the group V semimetals (phosphorus, arsenic and antimony) [52]. As shown in Figure 2a,b, the crystal structure of Bi exhibits typical layer stacking with *c*-axis layer spacing of 3.95 Å. The interlayer spacing of Bi is much larger than the radius of many alkali elements, including Li, Na, K and Mg, demonstrating its application potential for rechargeable alkali ion batteries.

### 2.2. Physicochemical Properties of Bi

Bi has been classified as a semimetal that has an electronic structure very different from that of other metal elements. Thus, Bi is endowed with many unique properties owing to its special electronic structure. Bi has a highly anisotropic Fermi surface and ultrasmall band overlap energy [56]. Bi has a theoretical conductivity value of 7.75 × 10^5^ S m^−1^ [57]. Thermodynamically, Bi can alloy with many alkali metals, including Li, Na, K and Mg, and obtain various bimetal alloying phases [51,53,54,55]. As shown in the Li-Bi alloy phase diagram, Bi can alloy with Li, forming different alloy phases, including LiBi, Li_2_Bi and Li_3_Bi (Figure 2c). Similarly, Bi can alloy with Na to obtain NaBi and Na_3_Bi (Figure 2d), alloy with K to obtain KBi_2_, K_3_Bi_2_ and K_3_Bi (Figure 2e), and alloy with Mg to obtain Mg_3_Bi_2_ (Figure 2f), respectively. As reported by Weppner and Huggins, the thermodynamic enhancement factor of Li-Bi alloy increases smoothly on the stoichiometry with a maximum of 360. The diffusion coefficient of Li ions in Li-Bi alloy is above 10^−4^ cm^2^ s^−1^ and also increases with the content of Li. Therefore, Bi has the potential as an anode material for LIBs, SIBs, PIBs and MIBs. Moreover, Bi has been used as a photocatalyst owing to its semimetal and plasmonic characteristics. Therefore, it is worth looking forward to its application as an electrocatalyst in the catalytic conversion reaction of sulfur cathodes.

## 3. Application of Bi-Based Composites for Energy Storage Systems

### 3.1. Anode for Alkali Ion Batteries

Commercial LIBs with graphite as anodes suffer from low energy density and are gradually approaching their fundamental limits, which cannot meet the demands of the energy storage market. Commercial graphite anodes for LIBs can only provide relatively low theoretical specific capacity, which further restrains the energy densities of commercial LIBs. Nowadays, the energy storage market’s requirements for energy storage systems are high specific energy density, high safety and stable cyclability. To meet the growing demands, some next-generation rechargeable LIBs and beyond-lithium ion batteries have been proposed. There are several criteria for the development of these novel energy storage devices, but the construction of high-performance electrode materials is a top priority of this technology. To achieve these targets, various anode materials based on insertion, conversion and alloying reaction mechanism are being explored and developed to meet high criteria for next-generation energy storage systems. In this context, bismuth is a typical alloy-type anode that can alloy with various alkali metal elements. As Table 1 illustrates, Bi-based anodes deliver high specific capacity and low operation voltage in LIBs, SIBs, PIBs and MIBs. However, the higher specific capacity is also accompanied by huge volume expansion due to alloying with multi alkali atoms. The volume expansion ratio was calculated by dividing the volume of the alloyed phase by the original volume of Bi. 

In order to alleviate the damage to the electrode material structure caused by volume expansion, various strategies have been explored and applied, including the hydrothermal/solvothermal method, electrospinning, dealloying, annealing, etc. Of those, the hydrothermal/solvothermal method is an effective method to synthesize nanomaterials with unique structures. Electrospinning techniques are usually applied to synthesize Bi-based precursors embedded in 1D nanofibers, followed by an appropriate pyrolysis process to obtain final Bi-based anode materials. Dealloying is an effective method for Bi-based nanomaterials with different dimensional structures [58]. The annealing process is a common synthesis method for Bi/C composites. Owing to the unique layered crystal structure of Bi, ultrasonic techniques are often used for the synthesis of 2D Bi nanosheets. The typical synthesis and preparation of Bi-based anode materials will be discussed in detail in the following sections. Herein, we summarize the development process for bismuth-based materials in alkali metal ion batteries and demonstrate researchers’ methodologies for the modification of Bi-based anodes.

#### 3.1.1. Li Ion Batteries

In recent decades, LIBs have spurred extensive investigations into mechanisms and applications in energy storage devices, and graphite has become the most widely used anode material for commercial LIBs. Its low theoretical specific capacity, however, restrains the energy density of current LIBs. It is difficult to meet the growing requirements for fast-charging, low-temperature operation and other extreme working conditions [59,60,61]. Hence, investigations for high-performance and safe anode materials for LIBs are imperative. A batch of materials have been investigated as promising anodes for next-generation LIBs. Li_4_Ti_5_O_12_ has flat discharge/charge plateaus around 1.55 V vs. Li/Li^+^, thus enabling circumvention of the formation of dendritic Li and dramatically improving system safety [62]. Nevertheless, the low theoretical specific capacity and low electric conductivity have seriously hindered its development. The specific capacity of silicon is about 10 times greater than that of graphite [63]. Nonetheless, Si-based anode suffers from large volumetric expansion and insulating properties. The low lithiation voltage makes it imperative to inhibit the dendrite growth of Li during fast charging processes [64]. Bismuth, with a high volumetric specific capacity (3800 mAh cm^−3^) and suitable lithiation potential (0.8 V vs. Li/Li^+^), is an ideal anode material with high-energy density. Moreover, the alloyed product of Bi, Li_3_Bi, has very high Li-ion conductivity, which is even comparable with fast solid ionic conductors and holds great promise for high-rate performance [65]. However, the electrochemical performance of Bi-based electrodes is hindered by its high volumetric expansion during alloying reactions and the unstable SEI films induced by side reactions at the interface of the electrolyte and electrode. To address these challenges, various modification methods have been introduced into Bi anodes, including carbon matrix modification, nanosizing and electrolyte engineering [66,67,68].

##### Modification Strategies

**Figure 3:**
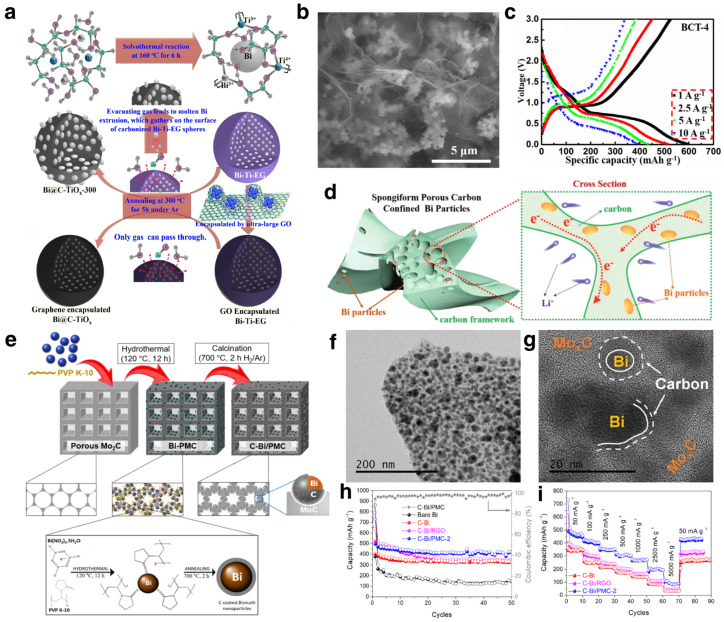
(**a**,**b**) Synthesis illustration with TEM image of Bi@C-TiO_x_; (**c**) voltage profiles of the Bi@C-TiO_x_ anode at current densities from 1 to 10 A g^−1^. Reproduced with permission [71]. Copyright 2018, Elsevier. (**d**) Illustration of the reaction mechanism of Bi/C composite. Reproduced with permission [72]. Copyright 2021, Wiley-VCH. (**e**–**g**) Schematic illustration and TEM images of the synthesis of C-Bi/PMC composites; (**h**) cycling performance of the C-Bi/PMC composites at 0.05 A g^−1^; (**i**) rate capability of the C-Bi/PMC composites at current densities from 0.05 to 5 A g^−1^. Reproduced with permission [73]. Copyright 2022, Elsevier.

An investigation of Li-Bi alloy was carried out by Weppner and Huggins in 1977 [65]. A galvanostatic intermittent titration technique (GITT) was employed to investigate the physicochemical characteristic of Li_3_Bi. The results indicate that Li-Bi alloy has a chemical diffusion coefficient of 10^−4^ cm^2^ s^−1^ in the high lithium deficit state that increases rapidly near the ideal stoichiometry. The lithiation process for Bi as an anode in LIBs contains a two-step alloying reaction, in which the intermediate phase is LiBi and the final product is Li_3_Bi. The reaction equilibrium potential of both is near 0.8 V vs. Li/Li^+^. Phase transformation caused by the alloying reaction induces volumetric expansion in the lithiation phase and severe internal stress in the boundary between the phases. The volume variation from Bi to LiBi is about 31%, and the volume further expands by 59% after final lithiation to Li_3_Bi. The severe volume variation will cause pulverization of the Bi-based electrode and exposure of fresh surface, inducing further consumption of electrolyte and forming fresh SEI films. In order to prevent active materials from losing electrical contact due to being wrapped by SEI films, a conductive substrate was used to form a composite with Bi. The modification with carbon materials was used in as early as 2014 to address the severe volume expansion and resulting structural damage [27]. A glucose-derived carbon shell can relieve the volumetric stress and accommodate the volume expansion. Therefore, mesoporous Bi@C composites with typical core-shell structure deliver enhanced cycling stability and rate capability. Since then, various synthesis methods have been developed to prepare many different types of Bi-C composites, including an aerosol spray pyrolysis technique [69], carbonization of Bi-metal organic frameworks [66], and pyrolysis of Bi-precursors [70]. Huang et al. proposed a surface engineering strategy and fabricated a Bi@C-TiO_x_ encapsulated by graphene nanosheets (Figure 3a) [71]. The structure of C-TiO_x_ confined Bi effectively buffers and suppress the volumetric variation of Bi. The large graphene wrapper further improves the electric conductivity and alleviates the volumetric stress of the Bi-based composites, as shown in Figure 3b. Owing to the well-designed nanostructure, the Bi-Ti-EG delivers remarkable rate capability at 10 A g^−1^, as shown in Figure 3c. Hong et al. constructed a spongiform porous Bi/C composite, in which the Bi nanodots are uniformly confined by functional carbon [72]. During an electrochemical reaction, the porous carbon provides a continuous conductive framework for electron shuttling. In addition, the spongiform porous structure with large specific area can enlarge the contact area of Bi-based anode in electrolyte and accommodate the volumetric variation during the charge/discharge process with abundant porous structure. The nanosizing Bi dots accelerate the diffusion process of Li ions, which effectively enhances the rate capability of the electrode (Figure 3d). It is well known that carbon materials, especially porous carbon materials, have low density. In some cases, adopting carbonaceous materials with low density could curtail the significant advantages of Bi-based materials as high-energy-density anode materials. For this reason, Devina et al. introduced hierarchically porous molybdenum carbide into Bi-based composites, due to the high energy density, mechanical stability and superior conductivity of the molybdenum carbide (Figure 3e) [73]. In as-fabricated C-Bi/PMC composites, the average diameter of the Bi nanodots is 6.4 nm (estimated by the Scherrer formula). As shown in Figure 3f,g, Bi nanodots with a carbon layer formed by heterogeneous nucleation and large particles formed by homogeneous nucleation are uniformly embedded on porous molybdenum carbide. Ultrasmall Bi nanodots provide a short diffusion path for Li ions. Then, the thin carbon layer and the porous molybdenum carbide support enhanced the electronic conductivity and released the volumetric stress induced by the alloying process for Bi upon cycling. Owing to the rational structural design, C-Bi/PMC delivers significantly higher reversible capacity and capacity retention than that of the control group (Figure 3h,i). Reviewing the published literature, modifications with carbon materials and other conductive matrixes, which are often accompanied by a decrease in particle size, can significantly boost the electrochemical performance of Bi-based anodes. On one hand, the conductive matrix in these structures can provide robust structural stability in the face of severe volumetric stress and avoid structural destruction and inactivation of active substances. On the other hand, the nanosized active substance can effectively accelerate the diffusion process and deliver outstanding rate performance of the electrode. The synthesis methods for various Bi-based anode materials and their corresponding electrochemical performance are presented in Table 2. Except for the investigations on half cells, Sadan et al. assembled a Li-ion full cell, with Bi powder as anode and LiFePO_4_ as cathode, that delivered the best electrochemical performance with stable cycling performance for 200 cycles in modified electrolyte [74]. 

##### Mechanism Investigations

**Figure 4:**
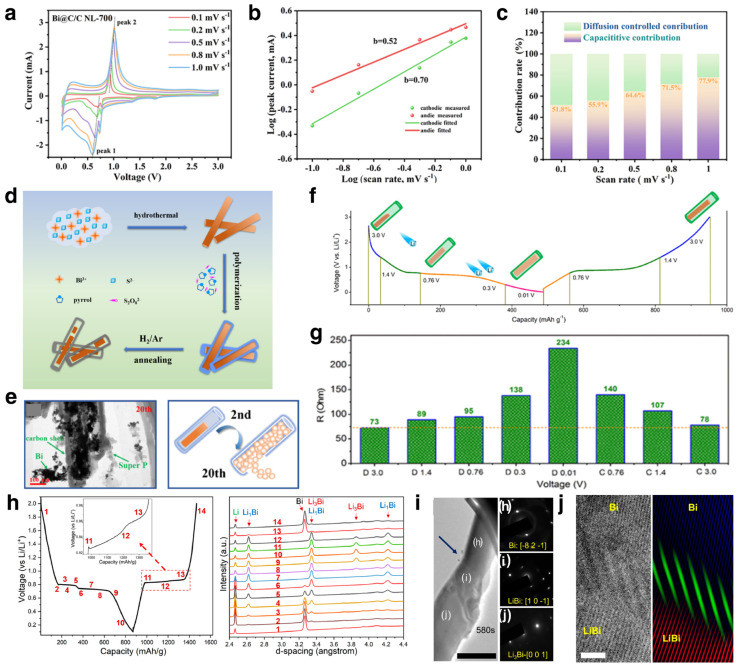
(**a**) CV curves of the Bi@C/C NL at different scan rates; (**b**) the corresponding b-value; (**c**) capacity contributions at different scan rates. Reproduced with permission [77]. Copyright 2023, Elsevier. (**d**) The synthesis schematic illustration of the Bi@C-N composites; (**e**) the structural change of the Bi@C-N from 2nd cycle to 20th cycle. (**f**,**g**) Voltage profile and the value of R of the Bi@C-N anode at different voltage points. Reproduced with permission [76]. Copyright 2019, American Chemical Society. (**h**) The in situ synchrotron XRD analyses based on Bi nanowires; (**i**) the TEM images of lithiating Bi nanowire and the SAED patterns of corresponding region; (**j**) HAADF image and the relevant IFFT of lithiating Bi nanowire. Reproduced with permission [78]. Copyright 2020, American Chemical Society.

In the research process for bismuth-based anode materials, in addition to continuously optimizing the material structure through various novel methods, it is also very important to study its electrochemical reaction mechanism through various advanced characterization methods. The battery system is a “black box” in which it is difficult to observe and investigate the structural evolution and phase transformation of the electrode materials. Researchers usually apply electrochemical characterization strategies for the electrochemical reaction kinetics of the electrode materials. CV tests at different scan rates (Figure 4a), EIS and GITT were often used to characterize the reaction kinetics via appropriate formula calculation [72,74,77]. According to the reported investigations, the peak current of the CV curves can be assigned to the capacitance and diffusion process. After calculated by the formula $i=av^{b}$, the capacity contribution of the capacitance and diffusion process can be unveiled. For most Bi-based nanomaterials with large specific area, the b-values are between 0.5 and 1, which means the capacity is contributed by both the capacitance and diffusion process (Figure 4b). With further calculation, the capacitance capacity contribution, which can be calculated quantitatively, often increases with the increase in scan rate (Figure 4c) [73,77]. 

Furthermore, some advanced in situ characterization techniques have been developed to unveil the phase transformation and structural evolution of the electrode materials with real-time observation. For instance, Hong et al. fabricated N-doped carbon coated Bi nanorods with yolk-shell structure (Figure 4d) [76]. Based on this, the failure mechanism of typical Bi-C composites was unveiled by ex situ TEM. During the first discharge process, the inner Bi nanorods undergo expansion and fragmentation and attempt to overflow. After 20 cycles, part of Bi particles overflow and are wrapped by SEI film, which may result in the rapid fading of capacity (with a capacity retention of about 63.1%) (Figure 4e). Electrochemical impedance spectroscopy (EIS) is a non-destructive testing method. The electrochemical impedance of the anode at different states of charge/discharge was tested and analyzed (Figure 4f,g). The calculated diffusion coefficient of Li^+^ increased during the primary lithiation process, mainly due to the loose structure. Then, the diffusion coefficient decreased as more Li^+^ ions were alloyed with Bi to form Li_3_Bi. The electrochemical impedance increased during lithiation and recovered after delithiation, demonstrating outstanding invertibility of the batteries. Until now, the volumetric variation of Bi-based anode was considered as the main reason for the structural failure in LIBs. Yuan et al. employed operando XRD and TEM techniques to unveil the phase transformation of single-crystalline Bi nanowires and proposed different structural failure mechanisms [78]. The operando XRD results demonstrated that the Bi nanowire anode undergoes a two-step phase transition, including Bi−Li_1_Bi and Li_1_Bi−Li_3_Bi, respectively, as shown in Figure 4h. The stepwise alloying reaction can be described by the following formula:

(1) $$Bi+Li^{+}+e^{-}↔LiBi$$

(2) $$LiBi+2Li^{+}+2e^{-}↔Li_{3}Bi$$

The in situ TEM illustrates that the surface lithiation will induce the precipitation of some nanograins with diameter of about 10 nm. The following lithiation of bulk Bi exhibits obvious diameter expansion. The enlarged view of the partially lithiated Bi nanowire shows a three-phase coexistence phenomenon, which is evidenced by selected area electron diffraction (SAED) characterization. The enlarged view of the Bi/Li1Bi two-phase boundary shows a Moiré pattern, indicating that the crystallographic orientation of the two-phase boundary is related to the Bi substrate (Figure 4i,j). This investigation demonstrates that surface engineering is important to improve the electrochemical performance of the Bi-based anode.

#### 3.1.2. Na/K Ion Batteries

Over the past three decades, LIBs have dominated market for portable electronics and electric vehicles owing to outstanding electrochemical performance. At the same time, the market’s energy storage demand also has been growing rapidly, including the popularity of electric vehicles and the construction of large-scale energy storage facilities. Due to the uneven global distribution of lithium resources, the rapid growth in demand has also led to rising lithium salt prices. SIBs and PIBs operate under reaction mechanisms similar to those of LIBs and have been considered as promising alternatives for LIBs [79,80]. Similarly to LIBs, the construction of electrode materials is also crucial to the advancement of SIBs and PIBs. The anode materials reported for SIBs and PIBs can be classified based on the reaction mechanism, including intercalation, conversion and alloy type. The intercalation-type anodes mainly include graphite, hard carbon, and Ti-based oxides, which usually exhibit good cycling stability owing to minimal volume change during sodiation and potassiation. Nevertheless, the low working voltage makes it easy for sodium/potassium metal nucleation and dendrite growth, causing severe safety risks. Additionally, the theoretical specific capacities of intercalation-type anodes are usually insufficient to satisfy demand for high-energy-density devices. Conversion materials mainly include transition metal oxides, chalcogenides and phosphides. Such materials are featured by low electronic conductivity, relatively high operating voltage and large volume change, leading to sluggish reaction kinetics and low power density. Alloy-type materials mainly include P, Sn, Sb, Bi, and Ge, which have suitable operating voltage and high specific capacity [81,82]. Bi can also be used as an anode material for SIBs and PIBs based on alloying reaction and delivers high volumetric specific capacity (3800 mAh cm^−3^). However, due to the larger radius of Na/K ions, the volumetric variations induced by alloying reaction in SIBs and PIBs are much more severe than in LIBs. Therefore, the requirements for the structural design of bismuth-based materials are more stringent. In recent years, a large amount of research has been invested in the modification of Bi-based materials mainly through structural engineering and electrolyte modification. In addition, the structure–property relationships, interfacial chemistry, and alloying reaction mechanisms of the Bi-based anode materials have been investigated through various progressive characterization techniques combined with theoretical calculations, and a series of breakthrough results have been achieved.

##### Modification Strategies for SIBs

**Figure 5:**
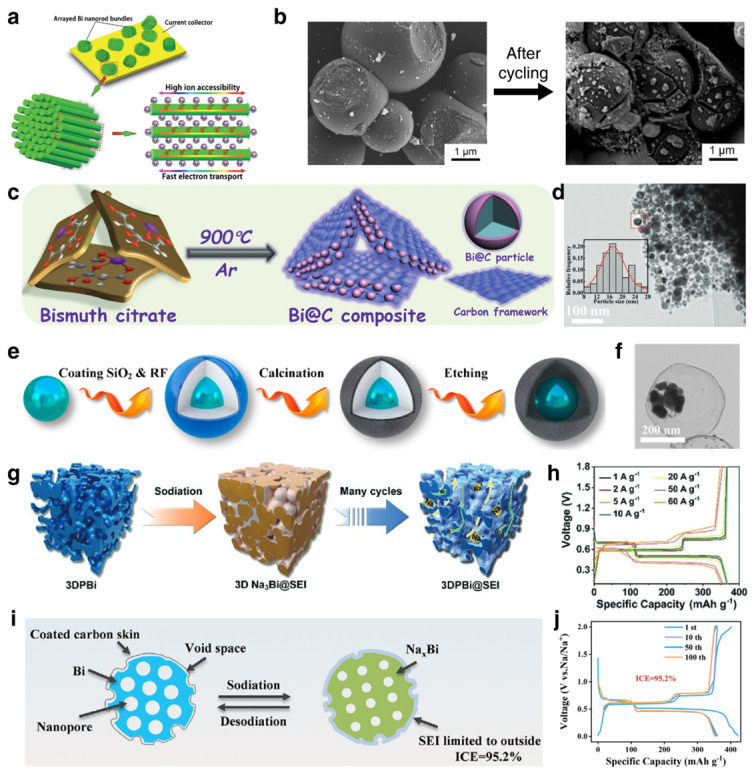
(**a**) Ion/electron transport of Bi nanorod anode in SIBs. Reproduced with permission [84]. Copyright 2016, The Royal Society of Chemistry. (**b**) SEM images of Bi microsphere before and after cycling. Reproduced with permission [26]. Copyright 2019, Royal Society of Chemistry. (**c**,**d**) Schematic illustration of the synthesis process and the TEM image of Bi@C composite. Reproduced with permission [86]. Copyright 2019, Wiley-VCH. (**e**,**f**) Schematic illustration of the synthesis process and the TEM image of Bi@void@C. Reproduced with permission [87]. Copyright 2020, American Chemical Society. (**g**) Illustration of the reaction mechanism of 3DPBi anode; (**h**) rate capability of the 3DPBi anode at current densities ranging from 1 to 60 A g^−1^. Reproduced with permission [90]. Copyright 2021, Wiley-VCH. (**i**) Illustration of the reaction mechanism of P-Bi/C; (**j**) voltage profile of the P-Bi/C anode in different cycles at 0.4 A g^−1^. Reproduced with permission [91]. Copyright 2023, Elsevier.

The investigation of Bi-based anodes in SIBs was first reported by Su et al. in 2015 with the Bi@graphene nanocomposite [23]. Due to the large interlayer spacing of the layered crystal structure of Bi along the *c*-axis, the sodium storage mechanism was demonstrated as an intercalation process, which was evidenced by an ex situ XRD technique. Combined with the research experience of LIBs, the structural modification strategy for Bi-based anodes mainly includes compounding with carbon materials, building alloy materials, nanosizing the particles and constructing porous skeletons. Zhao et al. fabricated Bi-Sb alloy via high-energy mechanical milling, and the composites delivered significantly enhanced cycling stability compared to single metal [83]. Liu et al. constructed arrayed Bi nanorod as enhanced anode material for SIBs [84]. The unique 1D nanostructured arrays with high ion accessibility and benign electronic conductivity delivered enhanced electrochemical performance in SIBs (Figure 5a). Cheng et al. observed the morphology of Bi microspheres before and after cycling via SEM characterization, as shown in Figure 5b. The Bi microspheres showed severe cracks after cycling due to the volume expansion and contraction during the discharge and charge process [26]. Additionally, some Bi-C composites were constructed via pyrolysis of Bi-based precursors and improved the electrochemical performance of Bi-based anodes [69,85]. However, the early researches about Bi-based anodes in SIBs were based on ester electrolytes. The nanosizing and composites with carbon materials improved the electrochemical performance to a certain extent, but it was not enough to meet people’s expectations. In 2017, Wang et al. coupled bulk Bi with an ether-based electrolyte (NaPF_6_-diglyme), which delivered surprisingly high electrochemical performance [28]. This study demonstrated that the bulk Bi anode undergoes morphological evolution from a bulk to porous structure after cycling in ether-based electrolyte, which is an interesting phenomenon and completely distinct from that in ester-based electrolytes. The derived porous structure can provide fast Na^+^ transport and superior structural stability, thus guaranteeing significantly enhanced electrochemical performance than that operating in ester-based electrolytes. The introduction of ether electrolytes has brought the development of bismuth-based anode materials to a new stage. To further enhance the cycling stability and rate performance, a carbon framework was introduced in Bi-based composite again by pyrolysis of the bismuth citrate (Figure 5c,d) [86]. Based on the ether electrolyte, the Bi@C composite gives an ultralong cycle life of 30,000 cycles when cycling at 8 A g^−1^ in SIBs. Yang et al. proposed a tunable yolk-shell structure with SiO_2_ as hard template and fabricated Bi@void@C nanospheres (Figure 5e,f) [87]. The optimized void space can effectively accommodate the volume change of the Bi core without unduly affecting the energy density of the battery. In addition, there are some other works based on the design of carbon coatings and reserved voids that have achieved better electrochemical performance [88,89]. In addition to providing pores through the carbon matrix to accommodate volume expansion, Cheng et al. designed a 3D continuous bulk porous bismuth (3DPBi) with interconnected voids between the Bi nanoligaments (Figure 5g) [90]. The porous Bi can accommodate the volume variation during repeated cycling processes with a stable structure and deliver unprecedented rate capability with a capacity retention of 95.6% at 60 A g^−1^ (Figure 5h). In this work, the initial coulombic efficiency was 65.9%, which was mainly due to the large specific area of the porous structure. Afterwards, Guo et al. construct a similarly porous Bi with carbon coating (P-Bi/C) [91]. The carbon shell blocks parasitic reaction between the highly reactive Na-Bi alloy and electrolyte, which induces a thin SEI film (Figure 5i). Owing to the well-designed structure, the initial coulombic efficiency reached 95.2%, as shown in Figure 5j. After years of research, reasonable structural designs and electrolyte engineering have made a qualitative leap in the electrochemical performance of Bi-based anodes. The typical samples of Bi-based anodes in SIBs with various synthesis methods and electrolytes are presented in Table 3, illustrating the significant effect of structural designs and electrolytes on the electrochemical performance. In addition to the investigations based on half-cells for the electrochemical performances of Bi-based anode materials, full-cell systems were built with appropriate cathodes for the exploration of practical applications of Bi-based materials. Wang et al. carried out a systematic study of a high-power Na_3_V_2_(PO_4_)_3_-Bi full cell system that can operate in a temperature range from −15 to 45 °C [92]. The full cell was assembled with bulk Bi as anode and CNTs-modified Na_3_V_2_(PO_4_)_3_ as cathode and operated in ether-based electrolyte, achieving high power density of 2.35 kW/kg^−1^ and energy density of 150 Wh/kg^−1^ (calculated based on the mass of both anode and cathode). Even operating at −15 °C, the full cell can still deliver a capacity of 234.9 mAh g^−1^, with a capacity retention of 84.3% in comparison to one operating at 40 °C. Except for conventional SIBs based on organic electrolytes, Bi-based materials also have been investigated for applications in aqueous SIBs in recent years. For instance, Zhu et al. developed a Bi-graphene composite via a laser-inducing method and used it as anode material for aqueous SIBs, which delivered impressive cyclability with a capacity retention of 122 mAh g^−1^ after cycling at a current density of 4 A g^−1^ over 9500 times [93]. Furthermore, the operating mechanism of Bi-based anodes in SIBs was systematically investigated and great progress achieved.

##### Mechanism Investigations for SIBs

**Figure 6:**
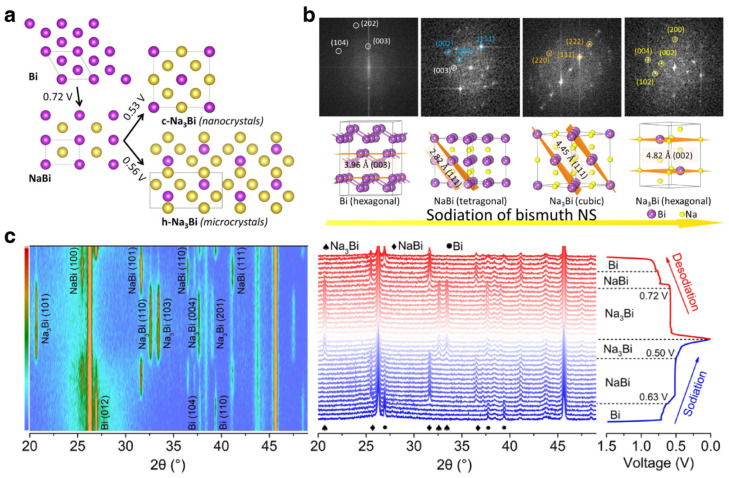
(**a**) Illustration of the different alloying reaction mechanism of Bi in SIBs. Reproduced with permission [102]. Copyright 2016, American Chemical Society. (**b**) The FFT patterns of in situ HRTEM images revealed structural evolution of Bi NSs. Reproduced with permission [103]. Copyright 2019, American Chemical Society. (**c**) Operando XRD of the HBiC anode in SIBs. Reproduced with permission [89]. Copyright 2021, AAAS.

In recent years, many advanced characterization techniques have been used to investigate the reaction mechanism of Bi-based anodes, including ex situ/in situ XRD and in situ HRTEM [87,101]. The understanding of the sodium storage reaction mechanism of Bi-based anode materials has also changed with the deepening of research. In 2014, Ellis et al. investigated Bi anodes in nonaqueous SIBs by in situ XRD and proposed a two-step alloying reaction process from Bi to NaBi and NaBi to Na_3_Bi. As mentioned earlier, Su et al. employed ex situ XRD measurements to study the reaction mechanism of Bi based on the Bi@graphene nanocomposite. The results demonstrated that the reaction between Na^+^ and Bi anode is an intercalation process rather than alloying process [23]. Then, Sottmann and coworkers proposed a size-controlled reaction path of Na-Bi alloy in non-aqueous metal ion batteries [102]. With the quasi simultaneous in operando synchrotron XRD/XAS characterization based on Bi/C composites, they proposed a different conclusion that the Bi anode with different particle size can lead to different Na_3_Bi phase, namely hexagonal Na_3_Bi (*h*-Na_3_Bi) and cubic Na_3_Bi (*c*-Na_3_Bi), as shown in Figure 6a. Bi with nanocrystalline material can form a metastable cubic polymorph of Na_3_Bi, which is conducive to the electrochemical behavior. Then, Huang et al. investigated the structure and phase transition of few-layer Bi nanosheets (Bi NSs) by in situ HRTEM [103]. By analyzing the fast Fourier transform (FFT) patterns of the HRTEM images of the alloy phase during the sodiation process, the transformation process (Bi→NaBi→*c*-Na_3_Bi→*h*-Na_3_Bi) was confirmed (Figure 6b). During this process, the *c*-Na_3_Bi was in a metastable phase, the *h*-Na_3_Bi was thermodynamic stable, and the existence of *c*-Na_3_Bi could buffer the severe structural variation from the *h*-Na_3_Bi. Therefore, the alloying reaction mechanism of Bi in SIBs can be summarized by the following formula:

(3) $$Bi+Na^{+}+e^{-}↔NaBi$$

(4) $$NaBi+2Na^{+}+2e^{-}↔Na_{3}Bi$$

Afterward, in situ XRD technology developed rapidly and has been used in the research of Bi-based anode materials and obtained clearer results [104]. For instance, Qiu et al. investigated the phase transform mechanism of a Bi-C hybrid framework derived from hierarchical metal-phenolic mesocrystal via in situ XRD, as shown in Figure 6c [89]. 

##### Modification Strategies and Mechanism Investigations for PIBs

**Figure 7:**
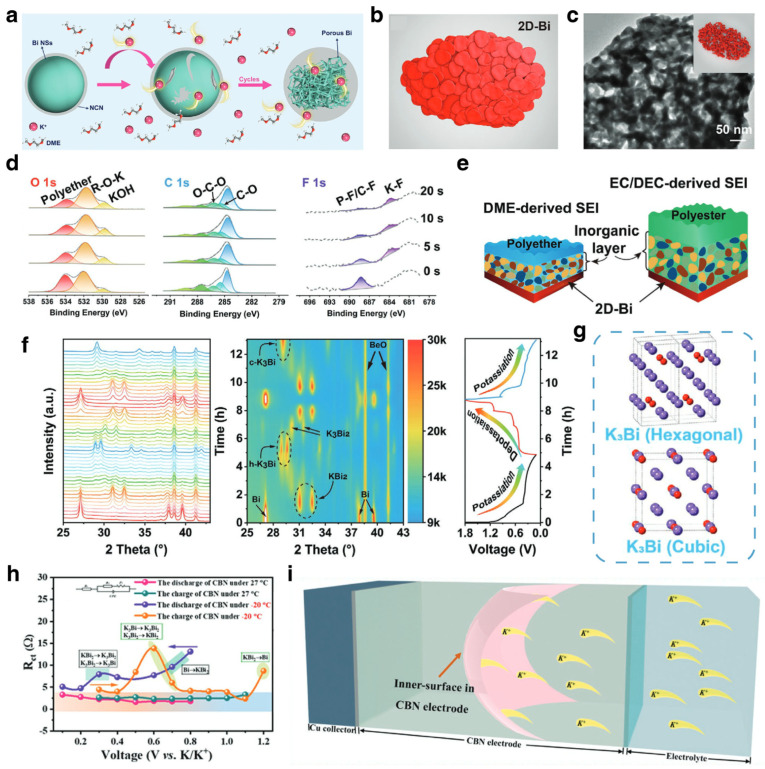
(**a**) Schematic illustration of morphological evolution of Bi NSs/NCNs. Reproduced with permission [106]. Copyright 2021, Wiley-VCH. (**b**,**c**) Schematic diagram of 2D-Bi and TEM image of the derived structure after first cycle; (**d**) in-depth XPS analysis of SEI of 2D-Bi after cycling; (**e**) illustration of the SEI structure of the 2D-Bi in different electrolytes; (**f**,**g**) operando XRD characterization of the 2D-Bi anode and the crystal structure of the K-Bi alloy. Reproduced with permission [99]. Copyright 2021, Wiley-VCH. (**h**) The R_ct_ of the CBN anode at different states of discharge/charge under 27 and −20 °C; (**i**) schematic illustration of the inner surface of the CBN anode. Reproduced with permission [100]. Copyright 2021, Wiley-VCH.

Based on the research progress in LIBs and SIBs, including performance optimization and mechanism research, Bi-based materials have been developing rapidly in potassium-ion batteries. Typical samples of Bi-based anodes in PIBs with various synthesis methods and electrolytes are presented in Table 3. Zhang et al. reported Bi/rGO nanocomposite as the anode for PIBs [24]. Electrolyte salt chemistry was investigated to boost the electrochemical performance of the Bi-based anode. The investigation revealed that the electrolyte configuration with KFSI is better than that of KPF_6_, as KFSI salt is versatile and can generate more stable SEI film during cycling. Also, for electrolyte modification, Zhang and coworkers chose ether electrolyte and proposed concentrated electrolytes with 5 M KTFSI in DEGDME for Bi-K batteries [97]. It was discovered that concentrated electrolytes can effectively passivate the surface of Bi-based anode, leading to high specific capacity, long lifespan and high coulombic efficiency. Lei et al. proposed a DME-based electrolyte for bulk Bi anode, which delivered superior cycling stability [47]. This work also presented the morphological evolution from bulk Bi into porous Bi after cycling, which is similar to what has been reported in sodium-ion batteries [28]. The derived porous structure of Bi can realize fast reaction kinetics and buffer the volume change during alloying and dealloying processes, leading to enhanced electrochemical performance. The alloying reaction mechanism of Bi anode in PIBs was uncovered by ex situ XRD with Rietveld refinement, which demonstrated a three-step alloying reaction, including Bi → KBi_2_ → K_3_Bi_2_ → K_3_Bi. After that, investigations into Bi-based anodes were mainly based on the ether electrolyte, and some innovative structural engineering methods were proposed for enhanced electrochemical performance [20,98,105]. Cui et al. fabricated a ball-cactus-like Bi encapsulated in N-doped carbon (Bi NSs/NCNs) via electrospinning followed by pyrolysis, which is applied for potassium storage [106]. In the initial state, bismuth exists as nanospheres and dispersed nanoparticles, but after cycling, it gradually transforms into porous bismuth, as shown in Figure 7a. By systematically comparing Bi nanomaterials with different dimensional structures, Cheng et al. found that two-dimensional bismuth (2D-Bi) evolved into the continuous porous Bi nanoligaments after cycling, as shown in Figure 7b,c. The porous nanoligaments can significantly shorten the diffusion path and buffer the volume expansion, leading to superior rate capability and stable cycling performance. Based on the 2D-Bi, the chemical structures of the SEI film in ester-based electrolyte and ether-based electrolyte were investigated by in-depth XPS, which demonstrated that the SEI film in the ether electrolyte was thin and tough, consisting of polyether and fluorine-containing compounds (Figure 7d). On the contrary, the SEI film in ester electrolytes was thicker and prone to rupture (Figure 7e). Additionally, the alloying reaction mechanism was also studied in this work by in situ XRD, and a new phase transform process was proposed. As shown in Figure 7f, Bi transforms into two intermediate phases, KBi_2_ and K_3_Bi_2_, and finally into hexagonal K_3_Bi (*h*-K_3_Bi) during the first potassiation process, and reversibly returns to the original state in the following depotassiation process. Notably, during the second potassiation process, the final product was cubic K_3_Bi (*c*-K_3_Bi) (Figure 7g), which demonstrated the existence of *c*-K_3_Bi in the K-Bi batteries. Therefore, the three-step alloying reaction of Bi in PIBs can be summarized by the following formula:

(5) $$2Bi+K^{+}+e^{-}↔KBi_{2}$$

(6) $$KBi_{2}+2K^{+}+2e^{-}↔K_{3}Bi_{2}$$

(7) $$K_{3}Bi_{2}+3K^{+}+3e^{-}↔2K_{3}Bi$$

The electrochemical reaction kinetics of Bi-based anodes in PIBs were also investigated via electrochemical characterization, such as CV and GITT. Common studies concluded that the capacity contribution of anode materials mainly includes diffusion and capacitive control [98]. As a supplement, Liu et al. proposed an inner surface-controlled mechanism in Bi-based anodes [100]. The in situ EIS test showed that the charge transfer resistances (R_ct_) of conjunct-like bismuth nanoparticles (CBNs) during different states of charge/discharge are stable and much lower than ever reported (Figure 7h). Combining the GITT and first-principle calculation, the diffusion coefficient (around 1.0 × 10^−9^ cm^2^ s^−1^) and migration barrier of K-Bi alloys (ranging from 22.18 to 44.36 kJ mol^−1^) were investigated, and an inner surface-controlled mechanism was proposed, which is similar to the electrochemical performance on the surface of pseudocapacitive materials (Figure 7i). Bi-based K-ion full cells were also investigated with appropriate cathode materials. Lei et al. combined bulk Bi anode with Prussian blue to form a K-ion full cell. The full cell operated in ether-based electrolyte and delivered an energy density of 108.1 Wh kg^−1^ with an average operating potential of 2.8 V [47]. Later, Cheng et al. assembled a K-ion full cell with modified Bi-based anode (2D-Bi) and Prussian blue that achieved an enhanced energy density of 174 Wh kg^−1^ and more stable cycling performance for 150 cycles [99]. Furthermore, the application of Bi-based materials in aqueous PIBs was also investigated. Qin et al. uncovered the mechanism of Bi anode in aqueous PIBs via ex situ XRD [107]. Bi anode in aqueous PIBs undergoes reversible alloying/dealloying reaction with K, which is different from the previously reported redox reaction mechanism. The Bi electrode delivered high specific capacity of 254.3 mAh g^−1^ and a long lifespan for 1600 cycles.

#### 3.1.3. Mg Ion Batteries

In recent years, MIBs have demonstrated enormous potential owing to their high gravimetric and volumetric specific capacity (2205 mAh g^−1^, 3833 mAh cm^−3^, respectively), abundant element reserves in Earth’s crust (2.3 wt%, ≈10^4^ times higher than that of Li) and environmentally friendly properties [108,109]. Additionally, compared with Li, Na and K, Mg anodes eliminate the risk of dendrite nucleation and growth under most operating conditions, leading to improved safety. Therefore, MIBs are viewed as very promising candidates among the next-generation energy storage systems. However, many challenges still need to be addressed for the development of pragmatic MIBs, especially the parasitic reaction between the Mg surface and common electrolytes. The insulating passivation layer formed on the metal anode will block the subsequent electrochemical reaction [110,111]. Although much effort has been invested in electrolyte modification, some shortcomings, including high cost, low stability and coulombic efficiency, still cannot be solved. Therefore, researchers have turned to looking for suitable electrode materials corresponding with appropriate structural modifications in the hope of achieving breakthroughs. Recently, alloy-type anodes for high performance MIBs have drawn widespread attention on account of high specific capacities, low operating voltage and electrochemical stability in conventional electrolytes. Among them, Bi can deliver relatively high volumetric capacity (1949 mAh cm^−3^ for Mg_3_Bi_2_). Moreover, Bi-based anode delivers a flat voltage plateau with low redox potential (~0.25 V vs. Mg^2+^/Mg). Nevertheless, some key challenges, such as large volume change and sluggish kinetics of Mg^2+^ during the alloying reaction process, still need to be overcome for greater advances. In this regard, great efforts have been made for high-performance Bi-based anode, such as reducing the size of Bi particles and compounding with carbon substrate. 

##### Modification Strategies

**Figure 8:**
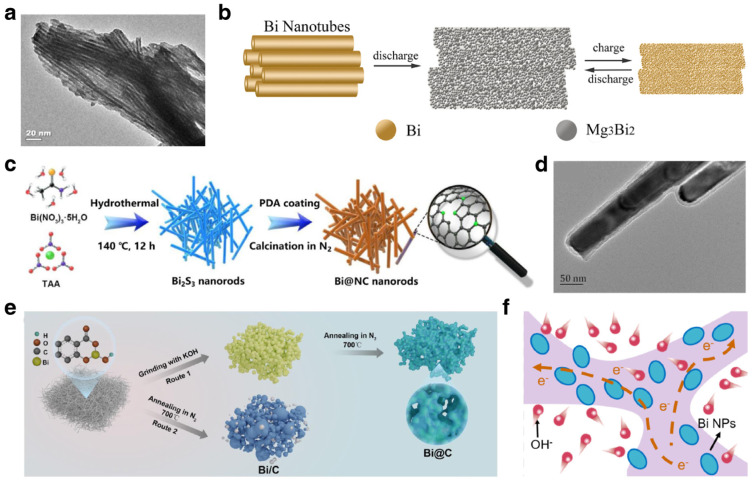
(**a**,**b**) TEM image and illustration of structural evolution of Bi nanotubes. Reproduced with permission [112]. Copyright 2014, American Chemical Society. (**c**,**d**) Synthesis process and TEM image of Bi@NC. Reproduced with permission [113]. Copyright 2021, Elsevier. (**e**) Synthesis process for Bi/C and Bi@C; (**f**) reaction mechanism of Bi@C anode. Reproduced with permission [117]. Copyright 2021, Elsevier.

Bi-based anodes were first reported as anodes of MIBs by Arthur et al. based on electrodeposited Bi film in 2012 [22]. This work demonstrated that the Bi anode delivered good cycling stability over 100 cycles. The Bi film anodes showed benign compatibility with conventional electrolyte, demonstrating promise for extending the voltage window for MIBs. The alloying reaction process with Mg_3_Bi_2_ as final product was evidenced by ex situ XRD results. Afterward, Shao et al. synthesized Bi nanotubes with uniform diameters of ∼8 nm (Figure 8a) that were used as high-performance anodes for MIBs [112]. The Bi nanotubes give high reversible specific capacity and high initial coulombic efficiency (95%). The morphological evolution during the discharge/charge process was investigated via TEM. As shown in Figure 8b, the Bi nanotubes are first converted to Mg_3_Bi_2_ nanoparticles, with the overall morphologies retained after discharge. In the following charge/discharge process, the magnesiation and demagnesiation will not cause structural collapse. Cen et al. proposed carbon-coated Bi nanorods (with average diameter of about 50 nm) as anodes for MIBs based on a low-dimensional design concept (Figure 8c,d) [113]. The core-shell Bi/C composite delivered outstanding electrochemical performance with 360 mAh g^−1^ at 0.1 A g^−1^ and long cycle life for 100 cycles. Additionally, some other Bi/C composites fabricated by annealing of appropriate Bi-based precursors also deliver enhanced electrochemical performance in MIBs [114,115,116]. As shown in Figure 8e, O-functionalized Bi nanoparticles embedded in porous carbon frameworks were used as high-performance anodes with remarkable capacity (347.2 mAh g^−1^ at 3 A g^−1^), excellent rate capability (a capacity retention 60% at 20 A g^−1^) and long lifespan (9500 cycles) [117]. The electrochemical reaction mechanism of the well-designed structure is shown in Figure 8f. This structure could buffer the volumetric variation and inhibit the aggregation of Bi in repeated magnesiation and demagnesiation processes. In the exploration of Mg-ion full cells, various cathode materials were applied in combination with Bi-based anodes for higher electrochemical performance. Shao et al. used Mo_6_S_8_ as a cathode to assemble an Mg-ion full cell, which operated in modified electrolyte and delivered stable cycling performance for 20 cycles [112]. Yu et al. assembled a Bi//MnO_2_ full cell in hybrid electrolyte, which delivered impressive electrochemical performance with operating potential of 1.62 V and a maximum energy density of 213 Wh kg^−1^ (calculated based on the mass of both Bi@C and MnO_2_) [117].

##### Mechanism Investigations

**Figure 9:**
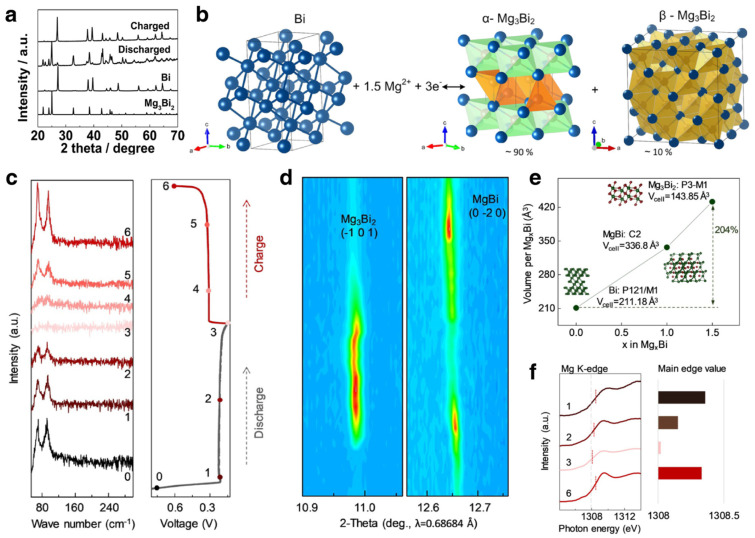
(**a**) XRD patterns of Bi nanotubes after charge/discharge. Reproduced with permission [112]. Copyright 2014, American Chemical Society. (**b**) Crystal structure of Bi and relevant phases after magnesiation. Reproduced with permission [118]. Copyright 2018, American Chemical Society. (**c**,**d**) Ex situ Raman and in situ XRD characterization of the p-Bi NS anode; (**e**) volume change rate of Bi on magnesiation; (**f**) NEXAFS spectra of the p-Bi NS. Reproduced with permission [119]. Copyright 2020, Wiley-VCH.

In addition to performance optimization, the operating mechanism of bismuth-based materials in MIBs has also been extensively studied via advanced characterization techniques. As shown in Figure 9a, Shao et al. investigated the Mg^2+^ insertion/deinsertion behavior via ex situ XRD. The XRD patterns of the Bi nanotubes before and after discharge/charge demonstrate the reversibility of alloying/dealloying reactions between Bi and Mg_3_Bi_2_ [112]. Kravchyk et al. combined crystal-structure prediction methodologies and total-energy DFT calculations to propose two stable phases, namely a low-energy trigonal phase (α-Mg_3_Bi_2_) and high-energy cubic phase (β-Mg_3_Bi_2_) (Figure 9b) [118]. Ex situ XRD measurements confirmed the existence of the trigonal and cubic Mg_3_Bi_2_ phases, where the cubic phase was the minor component (about 10%). In 2020, Xu et al. investigated the operating mechanism of Bi-based anodes via a variety of advanced characterization techniques, including operando XRD, NEXAFS and Raman (Figure 9c–f) [119]. According to the results, a reversible stepwise alloying reaction was proposed, which can be described by the following chemical reaction formula:

(8) $$Bi+Mg^{2+}+2e^{-}↔MgBi$$

(9) $$2MgBi+Mg^{+}+2e^{-}↔Mg_{3}Bi_{2}$$

Therein, the MgBi phase can deliver a volume buffer effect to lower mechanical disruptions. 

### 3.2. Modification for Alkali Metal Anodes

Alkali metal anodes have gained increasing attention in recent years owing to their high specific capacities (3861, 1165 and 678 mAh g^−1^ for Li, Na and K metals, respectively) and potential for high-energy-density secondary batteries [120]. However, metal dendrites can cause short circuits between the positive and negative electrodes, leading to thermal runaway and serious safety issues. Dendritic growth is a critical issue that must be eliminated to attain stable cyclability and dendrite-free metal anodes in a wide range of operating conditions [121]. Since the discovery of lithium dendrites, much effort has been invested in studying the growth mechanism of lithium dendrites and inhibiting dendrite growth. Many well-known reaction models have been established, greatly motivating the advancement of alkali metal batteries [122,123,124]. Meanwhile, many strategies have been proposed to inhibit dendrite growth and can be subdivided into electrode structure engineering, metal−electrolyte interfacial engineering and electrolyte optimization [125,126]. Cohn et al. employed anodes of SIBs, including carbon and alloy-type anodes, as the nucleation layers for anode-free batteries, demonstrating the significant effect of Bi on sodium metal anodes [49]. Recently, many reports have been released on the application of Bi-based alloys for the modification of alkali metal anodes, which will be discussed in detail in this section [127,128]. 

#### 3.2.1. Electrode Engineering

Compared with the alloy-type anodes of alkali ion batteries, bare alkali metal anodes have infinite volume variations during deposition and striping, which will cause serious damage to the electrode structure and inhomogeneous deposition and striping in subsequent cycling. Moreover, dendrite growth can be relieved by reducing the effective current density. Therefore, adopting an appropriate host with high surface area for alkali metal anodes is a promising strategy for lowering current densities and suppressing dendrite growth. To some extent, the host can provide a surface area several orders of magnitude larger than that of a planar current collector. Additionally, 3D hosts can accommodate volume expansion, promote uniform plating/stripping of alkali metals, and greatly improve the electrochemical performance of alkali metal anodes [129,130]. Therefore, extensive attention has been devoted to the modification of 3D hosts for alkali metal anodes. Nonetheless, common matrixes based on Ni, Cu and C are incompatible with alkali metals, which can cause severe polarizations and heterogeneous nucleation and deposition. Therefore, appropriate modification is necessary for constructing a suitable 3D host.

**Figure 10:**
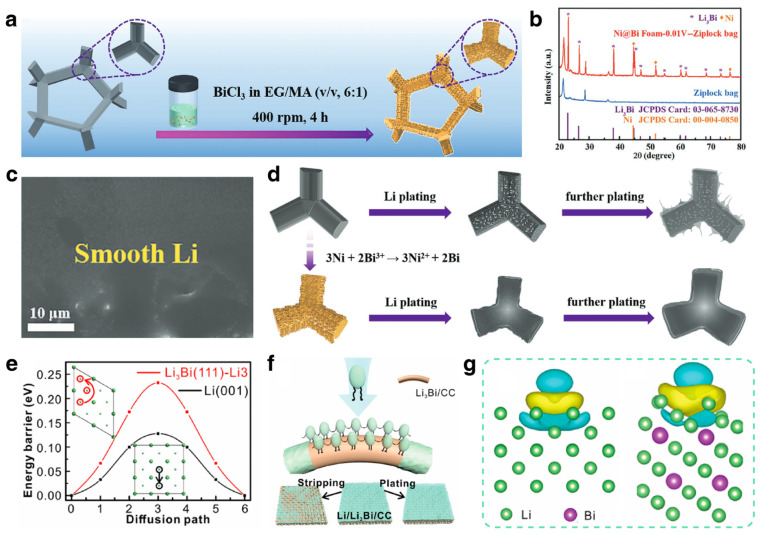
(**a**) Schematic illustration of the synthesis process for NBF; (**b**) XRD patterns of the NBF discharged to 0.01 V demonstrating the Li_3_Bi phase; (**c**) SEM images of NBF after plating with 5 mAh cm^−2^ Li; (**d**) schematic diagrams of Li plating behaviors on NF and NBF. Reproduced with permission [131]. Copyright 2021, Wiley-VCH. (**e**) Diffusion paths and energy barriers of Li on Li (001) and Li_3_Bi (111)-Li3; (**f**) schematic illustration of Li deposition on the Li_3_Bi/CC substrates; (**g**) electron density difference of Li on Li(001) and Li_3_Bi(111)-Li3 surfaces. Reproduced with permission [132]. Copyright 2021, American Chemical Society.

As for Li metal anodes, the alloying phase for Li_3_Bi is lithiophilic, which offers great potential for inducing homogeneous deposition/striping processes. Through a simple electrostatic displacement reaction, Bi-nanosheet-modified Ni foam (NBF) was constructed and used as a 3D host for a Li metal anode (Figure 10a) [131]. The Bi nanosheets on the Ni foam undergo in situ alloying reaction with Li and transform into a lithiophilic Li_3_Bi layer, as confirmed by the XRD results in Figure 10b. Compared with moss-like Li dendrites after plating on bare Ni foam, the morphology of the deposition on the NBF was smooth and dense (Figure 10c), demonstrating the effectively induced deposition. As illustrated in Figure 10d, the uniform Li_3_Bi nucleation sites can effectively inhibit the dendrite growth of Li and significantly improve the electrochemical performance. Furthermore, based on a lightweight and low-cost carbon matrix, bismuth oxide complex was constructed on carbon cloth and transformed into Li_3_Bi alloy via an in situ alloying reaction with Li metal, obtaining the 3D Li_3_Bi/CC scaffold [132]. Based on density functional theory (DFT) calculations, the operational principle of Li_3_Bi on suppressing dendrite growth was investigated. The diffusion energy barrier of the Li adatom on Li_3_Bi is 0.232 eV, which is higher than that of bare Li (Figure 10e). The high energy barrier illustrates high adsorption energy on Li_3_Bi, which efficiently restrains the dendrite growth (Figure 10f). The electron density difference plot demonstrates the strong interaction between Li and the lithiophilic substrate (Figure 10g). Owing to the reasonable design, the Li/Li_3_Bi anode delivered stable cycling performance at 20 mA cm^−2^ for 250 cycles. Apart from using Bi-based materials for modification of the 3D host, the construction of a Li alloy-composite also effectually suppressed the dendrite growth of Li metal anode material and significantly improved the electrochemical performance of LMBs [133]. Zhang et al. fabricated Li-Sn-Bi composite anodes to address the structural change [134]. Li_3_Bi alloy exhibits a skeleton structure to relieve the volumetric variation in the electrode and preserve its structural integrity during repeated cycling processes. Meanwhile, Li-Sn alloys can homogenize the plating process of Li metal. Thus, the alloy composite electrode delivered a lifespan of 2000 cycles at a high current density of 30 mA cm^−2^.

**Figure 11:**
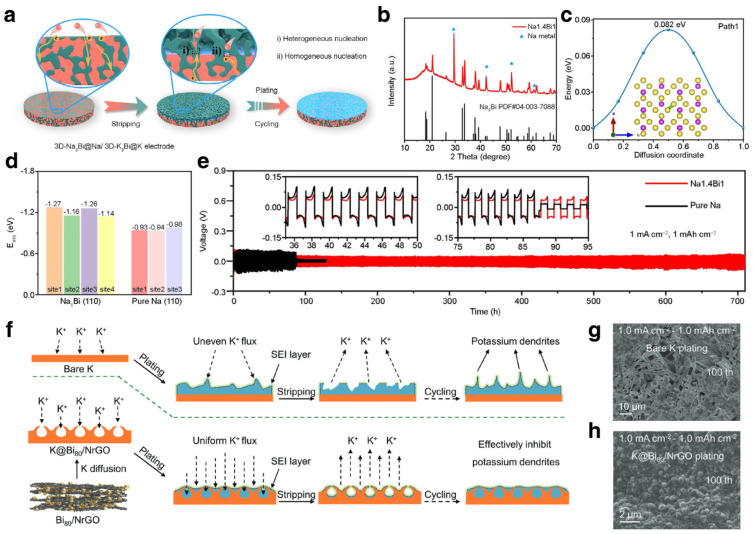
(**a**) Schematic illustration of the Bi-Na/K alloy; (**b**) XRD pattern of Bi-Na electrode; (**c**) diffusion energy barrier of Na through Na_3_Bi alloy; (**d**) adsorption energies of Na_3_Bi and pure Na; (**e**) voltage–time profiles of Bi-Na alloy and pure Na with 1 mAh cm^−2^ Na striping/plating at 1 mA cm^−2^. Reproduced with permission [137]. Copyright 2021, Elsevier. (**f**) Schematic diagram of potassium plating on bare K and K@Bi_80_/NrGO; (**g**,**h**) SEM images of potassium plating on bare K and K@Bi_80_/NrGO after 100 cycles. Reproduced with permission [48]. Copyright 2023, Wiley-VCH.

As for Na/K metal anodes, Bi can also be used to decorate the 3D matrix and construct alloy composites with Na/K metal. Xu et al. decorated porous Bi@C nanosheets on carbon cloth to serve as Na plating/striping hosts, which has merits of high surface area and favorable sodiophilicity [135]. The carbon substrate can render low local current density and homogenize the Na^+^ flux distribution. Owing to the Bi-based sodiophilic sites, homogenous nucleation and deposition can be guaranteed, leading to dendrite-free Na metal anodes. Moreover, alloy composites of Bi and Na/K were also constructed for dendrite-free metal anodes [136,137]. Ye et al. constructed a Bi-Na/K alloy to regulate the cycling performance of Na/K metal (Figure 11a,b). The DFT calculation results demonstrate the ultra-low diffusion energy barrier (0.082 eV) and high absorption energies of Na on the Na_3_Bi (Figure 11c,d). The well-designed metal anode ensures electric and ionic conductivity, which can effectively accelerate reaction kinetics and accommodate the volumetric variation on deposition and striping. When tested in a symmetrical cell, the 3D-Na3Bi@Na and 3D-K_3_Bi@K achieved stable cycling performance for over 700 h and 500 h, respectively. Feng et al. prepared a Bi-based self-supporting electrode (Bi80/NrGO) and used it as host for K metal [48]. The modified K metal anode (K@Bi80/NrGO) exhibited unique hollow pores that could accommodate potassium deposition and provide diffusion channels for K^+^. Further, the host could also regulate the electric field and K^+^ flux by abundant potassiophilic sites and thus achieve dendrite-resistant anodes (Figure 11f). The deposition morphology of the bare K surface showed growth of dendrites, whereas the deposited K@Bi_80_/NrGO was free of dendrites after 100 cycles (Figure 11g,h). Integrating experimental observation and multiphysics simulation, the hollow pore structure proved to be effective for regulating the K plating behavior. DFT calculations demonstrated that the Bi_x_K_y_ alloy possesses high binding energy with K atoms, which means the alloying Bi sites can anchor the K atoms and lead to homogeneous deposition.

#### 3.2.2. Interlayer Engineering

SEI film plays an important role in alkali ion batteries, as it determines the stability of the electrodes and cycle life of the batteries. As for alkali metal batteries, the SEI film is even more important, due to the high reactivity of alkali metal in common electrolytes that directly affects the reaction kinetics, cycling stability, and even safety performance of metal anodes. In alkali metal batteries, SEI film has multiple functions, including inhibiting further side effects, regulating ion flux, and inducing uniform metal deposition [138,139]. In principle, a desirable SEI film should have high mechanical toughness, high ionic conductivity, insolubility in electrolytes and thermodynamic and chemical stability. Therefore, interlayer engineering is extremely important to improve the electrochemical performance of alkali metal anodes. 

**Figure 12:**
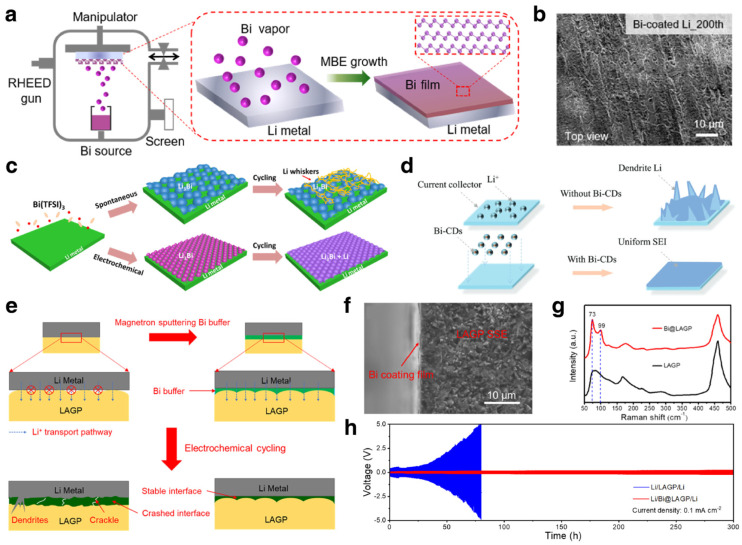
(**a**) The preparation process for ultrathin Bi film on Li substrate by molecular beam epitaxy; (**b**) SEM images of Bi-coated Li electrodes after 200 cycles. Reproduced with permission [144]. Copyright 2020, Elsevier. (**c**) Comparison of Bi layer formed by different reactions. Reproduced with permission [145]. Copyright 2022, American Chemical Society. (**d**) Schematic illustration of the effect of the Bi-CDs. Reproduced with permission [146]. Copyright 2022, Royal Society of Chemistry. (**e**) Schematics illustrating the ultrathin Bi buffer between solid-state electrolytes and Li metal; (**f**,**g**) SEM image and Raman spectra of the Bi buffer; (**h**) electrochemical performance of Li/SSE/Li symmetric cells when cycling at 0.1 mA cm^−2^. Reproduced with permission [147]. Copyright 2020, American Chemical Society.

In recent years, many strategies have been proposed to construct stable interlayers between metal anodes and electrolytes, including regulating the component and structure of SEI by adding electrolyte additives, employing high-modulus solid-state electrolytes, modifying the separator and constructing artificial protective layers [140,141,142,143]. Chen et al. deposited an ultrathin Bi film on Li metal by a molecular beam epitaxy technique, as shown in Figure 12a [144]. The deposited bismuth film undergoes an in situ alloying reaction with Li metal to form Li_x_Bi alloy phase, which can minimize the parasitic reaction between Li metal anodes and electrolyte. Further, the Li_x_Bi alloy layer provides abundant lithiophilic nucleation sites to guarantee homogeneous Li plating (Figure 12b) and rapid ion transport, realizing a dendrite-free Li metal anode with enhanced rate capability. Some Bi-based electrolyte additives were also used to construct stable interlayers for LMBs. Cui et al. introduced tetrapotassium heptaiodobismuthate (K_4_BiI_7_) as an additive into electrolyte [42]. The in situ constructed Bi-containing SEI layer effectively enhanced the stability of the Li metal anode. By introducing Bi(TFSI)_3_, a lithiophilic passivation layer was constructed on Li metal [145]. Moreover, the spontaneous reaction between Li and Bi(TFSI)_3_ can also generate a Bi layer, which was set as control group (Figure 12c). The SEM images and AFM test results after cycling demonstrate that the passivation layer formed by spontaneous reaction is loose and crackly. On the contrary, the electrochemically induced one is dense and smooth. Except for Li-Bi alloying dominant interfacial layer, Tu et al. applied Bi-doped carbon dots (Bi-CDs) as a surface modifier for stable Li metal [146]. The half-cell testing results showed high coulombic efficiency and a smooth surface, demonstrating that the increasing lithiophilic nucleation sites guarantee a uniform nucleation and deposition process for Li metal, as shown in Figure 12d. Owing to the Bi-CDs as interfacial modifiers, the Li metal anode stably cycled for 800 h. In addition to lithium metal batteries based on traditional liquid electrolytes, Bi-based materials are also used to improve the interface problem between solid electrolytes and lithium metal. Hu et al. constructed an ultrathin Bi buffer between Li_1.5_Al_0.5_Ge_0.5_P_3_O_12_ (LAGP) and a Li metal anode (Figure 12e) [147]. The Bi buffer significantly improved the physicochemical characteristic of the Li/SSE interface. As shown in Figure 12f,g, a SEM image of the cross-section and Raman spectra demonstrated that the Bi layer has a thickness of only 20 nm. Owing to the Bi buffer, the interface crash and dendrite growth were effectively suppressed. As shown in Figure 12h, the Li/SSE/Li symmetric cells with Bi buffer can stably cycle for 300 h with significantly reduced voltage hysteresis. 

**Figure 13:**
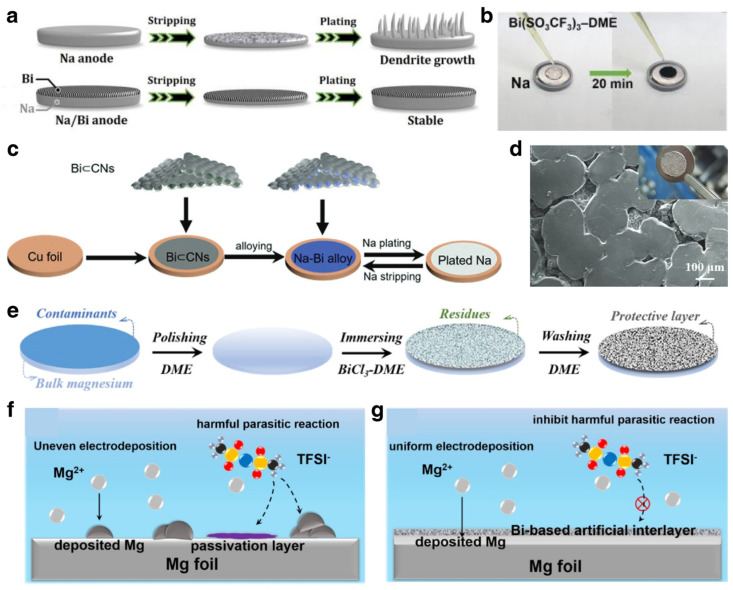
(**a**) Schematic illustration of deposition on Na and Na/Bi electrodes; (**b**) the synthetic process for Na/Bi anode. Reproduced with permission [148]. Copyright 2019, Wiley-VCH. (**c**) Schematic illustration of Bi⊂CNs and sodium plating/stripping processes; (**d**) optical and SEM images of Bi⊂CNs@Cu after 6 mAh cm^−2^ Na plating. Reproduced with permission [150]. Copyright 2021, Wiley-VCH. (**e**) Schematic illustration of the protected Mg foil; (**f**,**g**) schematic illustration of the electrochemical behavior of pristine Mg and protected Mg. Reproduced with permission [151]. Copyright 2021, American Chemical Society.

In addition to lithium metal batteries, Bi-based modified layers are also used to optimize the electrochemical performance of Na metal and Mg metal anodes. By dropping a solution of Bi(SO_3_CF_3_)_3_ and dimethyl ether (DME) on the surface of Na foil, a compact Bi layer was quickly constructed, which can effectively restrain the dendrite growth and inhibit the parasitic reaction (Figure 13a,b) [148]. The test Na metal with Bi layer showed a contact angle of about 0°, demonstrating better compatibility with the electrolyte. Electrochemical characterizations showed lower resistance and higher exchange current density of the Na/Bi anode. Owing to these merits, Na/Bi metal anode delivered stable cycling performance for 1000 h, which is about four times longer than achieved with bare Na. Inspired by the sodiophilicity of Na-Bi alloys, Bi-based materials were employed as robust nucleation buffer layers for dendrite-free Na metal anodes [149]. As shown in Figure 13c, Bi⊂CN composites were coated on a Cu current collector and used for Na deposition directly [150]. The optical and SEM images of the deposited Na metal showed uniform and island-like deposition morphology with a smooth surface (Figure 13d). As mentioned earlier, Mg metal anodes suffer from ionic insulating passivation films, thus resulting in irreversible electroplating/stripping behavior. Some works in the literature have reported the existence of fractal and dendritic Mg deposits, which is detrimental to safety. Zhao et al. reported a simple method for constructing Bi-based protective layer on Mg metal via immersing the Mg metal into a solution of BiCl_3_ and DME (Figure 13e). XRD results demonstrated the existence of Bi and Mg_3_Bi_2_ phases in the Bi-based layer. The protected Mg anode can stabilize for over 4000 cycles. As illustrated in Figure 13f,g, the Bi-based protective layer can effectively inhibit parasitic reactions between Mg metal and noncorrosive Mg electrolytes and induce homogenous deposition/striping of Mg during repeated cycling. 

### 3.3. Host for Sulfur Cathodes

Li-S batteries with merits of high specific capacity, low cost, and environmental friendliness have attracted considerable attention from researchers. Nonetheless, the shuttling effect of polysulfides and sluggish reaction kinetics seriously impact the electrochemical performance and commercialization of Li-S batteries. One of the effective solutions is to construct an appropriate structure by introducing an ideal sulfur host [152,153]. An ideal host for sulfur cathodes should have the following properties: strong polarity to anchor polysulfides, excellent electrocatalytic activity to accelerate the kinetics of polysulfide conversion reactions, large specific surface area to accommodate high sulfur loading and good conductivity to ensure the rapid progress of electrochemical reactions. In recent years, various catalysts have been developed for high-performance sulfur cathodes, such as metal oxides, sulfides, single-atom composites, etc. [154,155,156,157]. 

**Figure 14:**
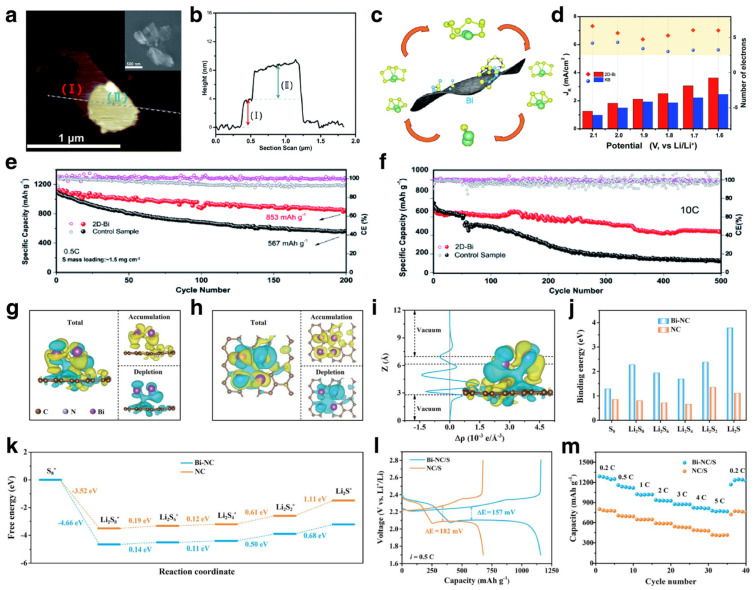
(**a**,**b**) AFM image and the corresponding height profile of 2D-Bi; (**c**) schematic illustration of the interior conversions on the 2D-Bi; (**d**) the reaction kinetic currents of the 2D-Bi and KB electrodes at the corresponding potentials; (**e**) the cycling performance of the 2D-Bi modified S cathode and control sample at 0.5 C. (**f**) Cycling performance of the 2D-Bi modified S cathode and control sample at 10 C. Reproduced with permission [50]. Copyright 2020, Royal Society of Chemistry. (**g**,**h**) Charge density difference of Bi-NC from side view and top view; (**i**) Δρ(z) of Bi-NC; (**j**,**k**) binding energies and Gibbs free energies calculation results for Bi-NC and NC; (**l**,**m**) voltage profiles and rate capabilities of Bi-NC/S and NC/S. Reproduced with permission [158]. Copyright 2021, Royal Society of Chemistry.

Bi has received widespread attention in the fields of photocatalysis, electrochemical nitrogen reduction, carbon dioxide reduction and other fields because of its unique energy band structure and catalytic conversion ability. Recently, some investigations of Bi-based materials have been carried out to solve the aforementioned problems, unveil the operating mechanism and improve the electrochemical performance of sulfur cathodes. Xu et al. prepared ultrathin bismuth nanosheets (2D-Bi) as an electrocatalyst for sulfur cathodes [50]. Atomic force microscopy (AFM) results demonstrated that the thickness of the 2D-Bi was about 4 nm (Figure 14a,b). First-principles calculations demonstrated that Bi has strong interaction with polysulfides due to the binding energies of polysulfides. Figure 14c illustrates the conversion reaction of polysulfides on Bi nanosheets. As shown in Figure 14d, the 2D-Bi electrode delivered a higher electron transfer number and higher current density in comparation with a Ketjen Black (KB) electrode. Therefore, with the help of 2D Bi, the utilization of sulfur is significantly improved, and the fast circulation of polysulfides and sulfur is guaranteed. Owing to the above merits, the 2D-Bi electrode delivers high reversible capacity and outstanding rate capability (Figure 14e,f). Except for the pure 2D-Bi, Bi/C composites with porous structure were also used for high-performance sulfur cathodes. Zhao et al. fabricated Bi/C composites (Bi-NC) for advanced sulfur hosts [158]. The differential charge density plot for the Bi-NC showed a charge shift in the heterogeneous interface, demonstrating the strong coupling relationship (Figure 14g,h). The planar-averaged electron density difference (Δρ(z)) further proved that the Bi induced local charge rearrangement, which is beneficial to strengthen the interaction with polysulfides (Figure 14i). The calculation results proved the higher binding energy of polysulfides on Bi-NC in comparison with those on NC (Figure 14j). The Gibbs free energies of reactions on Bi-NC and NC matrix demonstrated the enhancement of the reaction kinetics of sulfur cathodes (Figure 14k). As a result, the voltage curves of Bi-NC/S electrode showed lower potential polarization (Figure 14l). When cycling at 5 C, the Bi-NC/S electrode could still deliver a capacity of 779 mA h g^−1^ (Figure 14m). From the above, Bi-based materials have great application prospects for the modification of sulfur cathodes due to their adsorption effect and catalytic conversion capabilities for polysulfides. 

## 4. Conclusions and Perspectives

As reflected in the above discussions, due to their unique physical and chemical properties, the application of metallic Bi-based materials in the field of electrochemical energy storage, including alkali ion batteries, alkali metal batteries and Li-S batteries, has been widely reported. In this review, we have summarized recent advances in metallic Bi-based materials for these electrochemical energy storage systems from the perspectives of structural design, structure–property relationships, interfacial chemistry and reaction mechanisms. When used as anodes for alkali ion batteries, including LIBs, SIBs, PIBs and MIBs, Bi-based materials have high specific capacity and deliver outstanding electrochemical performance after various effective modification methods are employed, including reasonable structural designs, electrolyte optimizations and the formation of composites with appropriate supports. When used to optimize alkali metal anodes, the metallic Bi-based materials significantly improve the cycling stability and accelerate the electrochemical reaction kinetics of alkali metal deposition/striping processes. Through alloying with an alkali metal to form a corresponding alloy phase, the Bi-based alloy can effectively achieve a uniform distribution of ion flux and induce homogenous deposition and striping of the alkali metal, thus restraining dendrite growth and minimizing parasitic reactions between the alkali metal and electrolyte. When used as an electrocatalyst for sulfur cathodes, Bi-based materials also exhibit satisfactory effects and application potential. The high binding energy between Bi and sulfur species and the reduced Gibbs free-energy barrier ensure the outstanding performance of Bi-based sulfur cathodes. Therefore, metallic Bi-based materials have significant application potential in various energy storage systems. It will be necessary to further explore the optimal applications for metallic Bi-based materials and find advanced solutions to address unresolved key issues for next-generation electrochemical energy storage systems in the near future.

1. Some electrochemical performance indicators for Bi-based anodes in alkali ion batteries are still not fully satisfactory, including initial coulombic efficiency, performance of high-loading electrodes and energy density of the full cells. More efforts in structural and electrode design should be devoted to addressing these issues for practical applications. As mentioned earlier, metallic Bi-based materials will undergo particle refinement and morphological evolution during the first charge/discharge process, which will expose abundant fresh surfaces and lead to the continuous generation of SEI films. Therefore, reasonable structural design and electrolyte optimization will be beneficial to improving the initial coulombic efficiency. The porous structure will improve the cycle and rate performance of the batteries, but will also lead to a reduction in energy density. Therefore, it is necessary to find the optimal porosity and build a suitable porous structure.
2. The reaction mechanisms should be further investigated, including the morphological evolution mechanisms and alloying reaction mechanism. Judging from the published literature, metallic Bi-based materials tend to evolve into continuous porous structures after charge and discharge cycles. There is currently no clear explanation for this morphological evolution rule. The study of this rule will help to design more stable material structures. As for the final product and reaction pathways of the alloying reaction mechanism of Bi-based materials, there is currently no unified conclusion, and further research is needed.
3. In the application of metallic Bi-based materials for alkali metal anodes and sulfur cathodes, Bi plays a role as functional additive rather than active material. Therefore, the content of Bi-based materials can be reduced as much as possible for maximal effect. Electrode engineering for alkali metal anodes is a promising research direction for the development of a suitable and stable 3D skeleton structure with nanosized Bi uniformly dispersed to induce homogeneous deposition of metal ions. Interfacial engineering plays an important role in the alkali metal anode. The ideal metal anode interface layer should be composed of inorganic components with high mechanical strength and organic components with highly elasticity. Therefore, hybrid organic–inorganic SEIs with Bi-based components hold promise for better alkali metal anodes.

## Figures and Tables

**Table 1:** The characteristics of Bi-based anodes for alkali ion batteries.

Application	Products	Mass Specific Capacity (mAh g^−1^)	Volumetric Specific Capacity (mAh cm^−3^)	Operation Voltage (V)	Volume Expansion Ratio (%)
LIBs	Li_3_Bi	385	3800	0.8/0.7	208
SIBs	Na_3_Bi	385	3800	0.77/0.67	352
PIBs	K_3_Bi	385	3800	1.0/0.4/0.3	509
MIBs	Mg_3_Bi_2_	385	3800	0.25	196

**Table 2:** Lithium storage performance of Bi-based anode in recent reports.

Materials	Synthesis Methods	Electrolytes	ICE (%)	Cycling Performance ^(a)^	Rate Capability ^(b)^	Refs.
Bi@C nanowires	pyrolysis	1 M LiPF_6_ in EC/DEC/DMC	63.1	408/100th/0.1	240 (58.8)/1	[27]
Bi@C microspheres	aerosol spray pyrolysis	1 M LiPF_6_ in EC/DEC/DMC	36.6	280/100th/0.1	90 (30.1%)/2	[69]
Bi/C nanofibers	pyrolysis	1 M LiPF_6_ in EC/DEC	61.5	316.7/500th/0.1	159.3 (49.7%)/3.2	[75]
Bi/C nanowires	mechanical pressure injection method	1 M LiPF_6_ in EC/DEC	46.7	307.3/50th/0.2	-	[67]
Bi@C-TiO_x_	solvothermal	1 M LiPF_6_ in EC/DMC/EMC	-	119.5/5000th/10	225 (67.5%)/10	[71]
Bi/C	solvothermal	1 M LiPF_6_ in EC/DMC	52	248/100th/1	208 (26.1%)/2	[66]
yolk−shell Bi@C−N	thermal reduction and carbonization	1 M LiPF_6_ in EC/DEC/DMC	73	1700 mAh cm^−3^/500th/1	1635 mAh cm^−3^ (41.8%)/2	[76]
Bi@C	carbothermal reduction	1 M LiPF_6_ in EC/DEC with 10% FEC	64	256/1400th/1	131 (23.9%)/5	[70]
Bi@PC	carbothermal reduction	1 M LiPF_6_ in EC/DEC/DMC	61.8	380/500th/0.5	215 (30.1%)/2	[72]
C-Bi/PMC	annealing	1 M LiPF_6_ in EC/DMC with 1% VC and 5% FEC	68	400/500th/0.25	90 (21.3%)/5	[73]
Bi powder	commercial	2 M LiBH_4_ in THF	-	381/1000th/8 C	230 (56.8%)/64 C	[74]
Bi@C/C NL	pyrolysis	1 M LiPF_6_ in EC/DMC (3:7) with 5% FEC	62.3	373/1500th/3	278 (48.3%)/3	[77]

^(a)^ The cycling performance was recorded by specific capacity (mAh g^−1^)/cycle number/current density (A g^−1^); ^(b)^ the rate capability was recorded by specific capacity (mAh g^−1^) (capacity retention)/current density (A g^−1^).

**Table 3:** Sodium/potassium storage performance of Bi-based anodes in recent reports.

Materials	Synthesis Methods	Electrolytes	ICE (%)	Cycling Performance ^(a)^	Rate Capability ^(b)^	Refs.
Bi@graphene	hydrothermal	1 M NaClO_4_ in EC/PC	55.6	358/50th/0.04	250 (69.8%)/1.28	[23]
Arrayed Bi	dealloying	1 M NaClO_4_ in PC with 5%FEC	55	301.9/150th/0.05	102.3 (29.2%)/2	[84]
Bi@C	aerosol spray pyrolysis	1 M NaClO_4_ in EC/PC	36.6	123.5/100th/0.1	83.4 (32.1%)/2	[69]
Bi/CNF	electrospinning	1 M NaPF_6_ in EC/DMC	61.6	483.8/200th/0.1	170.7 (32.0%)/2	[94]
Bi/CFC	hydrothermal	1 M NaPF_6_ in EC/DMC/EMC with 5%FEC	61.2	350/300th/0.05	~100 (28.5%)/2	[95]
Bi-NS@C	molten salt calcination	1 M NaClO_4_ in EC/PC	-	106/1000th/0.2	110 (64.7%)/2	[96]
Bulk Bi	commercial	1 M NaPF_6_ in G2	94.8	389/2000th/0.4	356.0 (90.2%)/2	[28]
Bi/C nanofibers	electrospinning	1 M NaPF_6_ in EC/DMC with 5%FEC	55.8	273.2/500th/0.1	69.0 (22.8%)/3.2	[75]
Bi@Graphite	intercalation	1 M NaPF_6_ in DME	74.5	~140/10,000/3.2	113 (70%)/48	[18]
Bi@C Nanoplates	thermal treatment	1 M NaPF_6_ in EC/DMC with 5%FEC	69.1	200/200th/0.15	74 (24%)/2	[85]
Bi@3DGF	thermal treatment	1 M NaPF_6_ in DME	36	185.2/2000th/10	180 (78.3%)/50	[26]
Bi@C	annealing	1 M NaPF_6_ in DME	50.3	265/30,000th/8	232 (71%)/60	[86]
Bi@N−C	annealing	1 M NaPF_6_ in DME	85.7	302/1000th/1	368 (89.7%)/2	[88]
Bi/C	annealing	1 M NaPF_6_ in DME	36.5	203/1000th/10	178 (60%)/100	[25]
3DPBi	liquid phase reduction	1 M NaPF_6_ in DME	65.9	374/3000th/10	354 (95.6%)/60	[90]
HBiC	annealing	1 M NaPF_6_ in DME	79.9	264/15,000th/5	72.5 (22.9%)/200	[89]
P-Bi/C	annealing	1 M NaPF_6_ in DME	95.2	178/20,000th/50	101 (27.3%)/72	[91]
Bi/rGO	solution synthesis method	1 M KFSI in EC/DEC	63	290/50th/0.05	235 (76.1%)/0.5	[24]
bulk Bi	commercial	1 M KPF_6_ in DME	87.2	322.7/300th/0.8	-	[47]
Bi@C	carbothermal reduction	5 M KTFSI in DME	46.3	151/35th/0.1	-	[97]
FBNs	electrochemical cathodic exfoliation	1 M KPF_6_ in DME	-	201/2500th/20	182 (43.0%)/20	[98]
2D-Bi	solution synthesis method	1 M KPF_6_ in DME	89.2	344/750th/10	345 (87.3%)/30	[99]
CBN	solution synthesis method	1 M KPF_6_ in DME	-	200/5000th/30	254.5 (58.6%)/30	[100]

^(a)^ The cycling performance was calculated as specific capacity (mAh g^−1^)/cycle number/current density (A g^−1^); ^(b)^ the rate capability was calculated as specific capacity (mAh g^−1^) (capacity retention)/current density (A g^−1^).

## Data Availability

Data are contained within the article.
